# 

**Published:** 1889-07

**Authors:** 


### CONTENTS.

### COMMUNICATIONS.

President's Address—Meeting of M. D. A. .... 317
Cocaine Anæsthesia .... 323
Filling Root Canals of Human Teeth .... 333
What Shall We Do in the Case? .... 339
The Survival of the Unfit in Human Dentition .... 345
Restoring Articulation by Tipping Molars with Gold .... 347

### SELECTIONS.

Odontological Society of Great Britain .... 350
Methods of Instruction in Medicine .... 353
Cocaine in Teeth Extraction .... 354
Microzoa .... 355
Capping Exposed Pulps .... 357
Dental Fistulæ .... 357
Bacteria in Milk .... 358
International Dental Congress .... 359
Ideal for a Medical Society .... 362
University of Michigan .... 363

### EDITORIAL.

American Dental Association .... 364
American Medical Association .... 364
The National Association of Dental Examiners .... 366
The National Association of Dental Faculties .... 366
American Dental Association .... 367
To Saratoga .... 367
The First Matches .... 367
Obituary .... 368

